# Adenoid cystic carcinoma of breast and the importance of differentiation from collagenous spherulosis by FNAC

**DOI:** 10.4103/0970-9371.70748

**Published:** 2010-04

**Authors:** Amrish N Pandya, Pinal Shah, RD Patel, Prashant R Patel

**Affiliations:** Department of Pathology, Government Medical College and New Civil Hospital, Surat, Gujarat, India

**Keywords:** Adenoid cystic carcinoma, breast, collagenous spherulosis, FNAC

## Abstract

We are presenting a case of adenoid cystic carcinoma (ACC) of breast in a 66-year-old woman having lump in left breast, admitted to surgical ward of our institute. A diagnosis of ACC of breast was made and subsequently confirmed histopathologically and on immunohistochemistry.

## Introduction

Adenoid cystic carcinoma (ACC) comprised 0.1% of all breast carcinoma,[1,2] and are associated with an excellent prognosis. They are more commonly described in salivary gland where they have more aggressive course. In contrast to extramammary ACC, those arising in breast have an excellent prognosis.[3,4] It occurs predominantly in perimenopausal women. Clinically, the lesion is well circumscribed in sub-areolar or central region of the breast but nipple discharge is uncommon and cytologically cellular with uniform round hyperchromatic cells, present singly and in loosely cohesive clusters. Tumour cells surround cores and balls of acellular homogeneous material (hyaline globule).

It should be differentiated from benign conditions, such as collagenous spherulosis (CS) and other malignant conditions, such as invasive cribriform carcinoma.[5]

## Case Report

A 66-year-old woman presented to cytopathology laboratory of our institute with complaint of left breast lump since 3 years, which gradually increased to the present size. She had lumpectomy done on the left breast 20 years ago. Obstetrical history was not significant. She was menopausal for the last 20 years.

On examination the breast showed a well circumscribed 1.6×1.6 cm nodular lesion located in the sub-areolar region which was of firm consistency. The lesion was attached to the overlying skin and nipple retraction was seen. Ultrasonography was suggestive of a malignant mass lesion.

**Figure 1:**
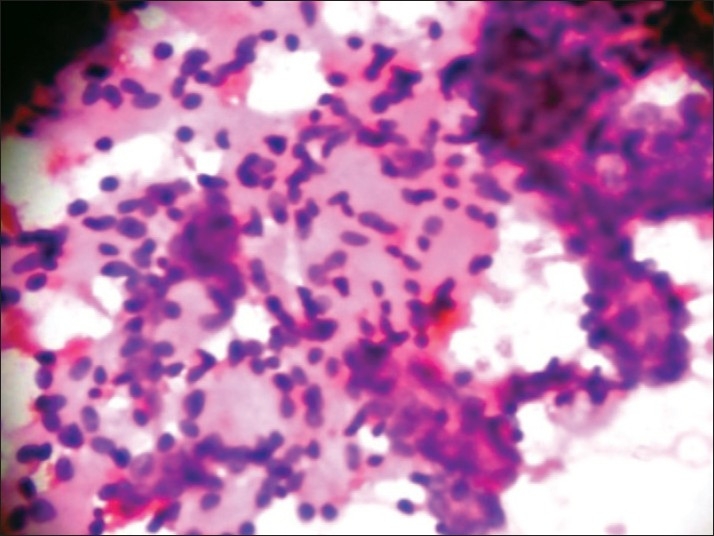
Cells arranged in microcystic pattern, with beaded fragments of hyaline stroma between cell clusters (H and E, ×400)

**Figure 2:**
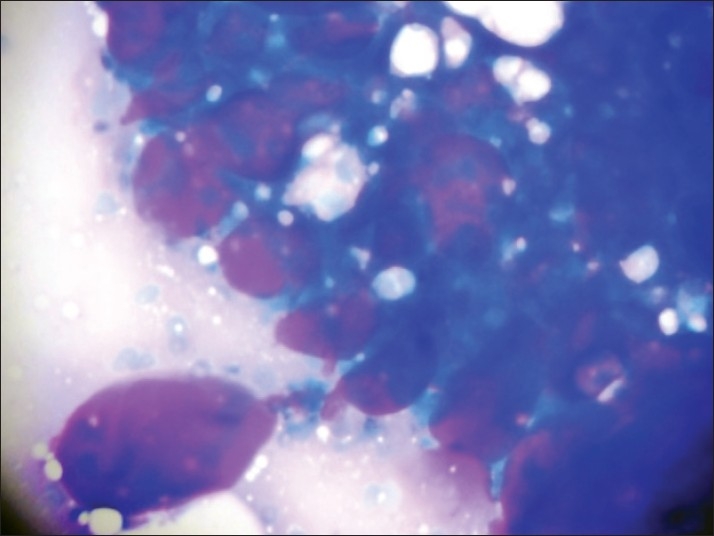
Hyaline stromal globules of variable size, appear magenta coloured (MGG, ×200)

Fine needle aspiration cytology was done using a 22-gauge needle and 10 mL syringe. Both alcohol-fixed and air-dried smears were prepared and stained by hematoxylin and eosin (H and E) and May-Grünwald-Giemsa (MGG) stains, respectively. The smears were moderately cellular with a relatively clean background, lacking inflammation or necrosis. The epithelial cells were arranged in a microcystic pattern, with some epithelial cells arranged in clusters and cup-shaped fragments. The cells had scanty cytoplasm with high nucleo-cytoplasmic ratio. The nuclei were uniform, small, round to oval and hyperchromatic [Figure 1]. Some epithelial cells adhered to hyaline stromal globules of variable size and appeared magenta colored in MGG stain [Figure 2] and light pink in H and E stain.

A diagnosis of ACC was made while considering cytological features along with history, age and location of the tumour. The differential diagnosis included CS. Excision was advised. Subsequently, histopathology confirmed the diagnosis.

## Discussion

ACC of breast is rare, and slow growing neoplasm accounting for 0.1% of all breast neoplasms. It has favorable prognosis, as lymph node involvement and distant metastasis are uncommon.[6] ACC of breast was first termed as “cylindroma” by Billroth. The tumour occurs predominantly in women with a mean age of 60–64 years. It is rarely bilateral, and the most frequent presenting symptom is tender breast mass, which was present in our patient. Pain is a symptom of ACC of salivary gland, attributed to perineural invasion, which is rare in ACC of breast. It is uncertain why tenderness is a predominant symptom in patients with ACC of breast with absent perineural invasion.[3]

Due to its excellent prognosis, cytological diagnosis is very important. It should be differentiated from benign conditions, such as CS on cytology. In our case, the cells of ACC were small, uniform with small, round hyperchromatic nucleus, scanty cytoplasm surrounding periodic acid Schiff (PAS)-positive hyaline globules and no bipolar nuclei.[7]

CS is a benign entity and mostly an incidental microscopic finding, rarely presenting as palpable mass occurring in the age group of 39–55 years. It is usually associated with other benign lesions, such as ductal hyperplasia, proliferative breast lesions, such as intra-duct papilloma, papillary duct hyperplasia, atypical ductal hyperplasia and sclerosing adenosis. On cytological examination, it shows epithelial cells in clusters, scattered singly, with myoepithelial cells and spherules that appear magenta coloured in MGG and light pink in H and E stained slides. Variable positive staining with periodic acid Schiff and Alcian blue stains and numerous bipolar nuclei in the background.[8,9]

## Conclusions

Cases of ACC may simulate CS. Cellular smear with uniform round hyperchromatic cells present singly and in loosely cohesive clusters, absence of bimodal cell population and tumor cells surrounding cores and balls of acellular homogeneous material (hyaline globule) clinches the diagnosis of ACC of breast. It is important to differentiate ACC from CS because of innocuous nature of the latter which does not need any intervention.
